# Stability and optical properties of plutonium monoxide from first-principle calculation

**DOI:** 10.1038/s41598-017-12428-x

**Published:** 2017-09-22

**Authors:** Ruizhi Qiu, Yongbin Zhang, Bingyun Ao

**Affiliations:** Science and Technology on Surface Physics and Chemistry Laboratory, 621908 Jiangyou, Sichuan P.R. China

## Abstract

The resolution of questions about the existence of condensed plutonium monoxide (PuO) has long been hindered by lack of thermochemical data. Here we perform first-principles calculation to investigate the reaction Pu_2_O_3_ + Pu → 3 PuO and find that PuO is thermodynamically unstable under ambient pressure. We also find that pressure could stabilize PuO by strengthening the hybridization between Pu-5*f*/6*d* and O-2*p* states. Moreover, the dynamical stability of NaCl-type PuO is verified by the phonon calculation. Optical properties such as reflectivity are also predicted for the detection of metallic PuO.

## Introduction

Despite the technological importance and scientific interest of metallic plutonium (Pu), its phase diagram with oxygen is still controversial. Now it is recognized that the metal is covered by a layer of trivalent sesquioxide (Pu_2_O_3_), followed by a layer of dioxide (PuO_2_)^1,2^. The existence of Pu_2_O_3_ is inevitable because the reduction of PuO_2_ by Pu (i.e., 3 PuO_2_ + Pu → 2 Pu_2_O_3_) is thermodynamically favourable^1^. In addition, PuO_2−*x*_ is shown to exist between PuO_2_ and Pu_2_O_3_^3^. However, the existence of plutonium monoxide (PuO) between Pu_2_O_3_ and Pu or in the bulk form has not been proven conclusively. Although several investigators reported its preparation including the surface^4–6^ and bulk phase^3,7–10^, there are also strong contradictory evidences^11–13^.

Sooner after the preparation of metallic plutonium in 1944, PuO was first observed by Zachariasen^4^ as a silver layer on the surface of plutonium metal, and it was identified using X-ray diffraction (XRD) as the NaCl-type structure with the lattice parameter being 4.948 ± 0.002 Å^4,14^. Another value of lattice parameter reported by Coffinberry and Ellinger was given as 4.96 ± 0.01 Å^3,5^. In 1960, Akimoto^7^ reported the preparation of PuO in the bulk form by the reaction of molten plutonium with the oxygen generated from Ag_2_O. The measured lattice parameter was given by 4.960 ± 0.003 Å. Later Chikalla *et al*.^3,8^. prepared PuO by reacting PuO_2_ and carbon at 1800°C in helium and at 1550°C in vacuum. However, Mulford *et al*.^11^. stated that all attempts to prepare PuO in bulk at Los Alamos have been unsuccessful. In addition, the authors found that the solid solution phase, Pu(C, O), could not exist with a high oxygen content and thus argued that PuO does not exist as a pure binary phase. In fact, Chikalla *et al*.^3,8^. already suspected that their compound may be a carbon-stabilized form or an oxycarbide. Furthermore, the high-temperature XRD study of Terada *et al*.^6^, which was performed on the PuO_2_-coated metal, showed that the formation reaction of PuO,

1 $$\,{\rm{P}}{\rm{u}}{}_{2}{\rm{O}}{}_{3}+{\rm{P}}{\rm{u}}\to 3\,{\rm{P}}{\rm{u}}{\rm{O}},$$

begins only at high temperature of about 250°C. The authors also noted that the free energy change for reaction (1) is positive as calculated from Oetting’s table^12^, i.e., PuO is metastable. The landmark experiment was performed by Larson and Haschke^13^, who used X-ray photoelectron spectroscopy (XPS) and Auger electron spectroscopy (AES) to identify the surface phase of vacuum-heated-treated PuO_2_-coated metal. They showed that the surface phase previously identified as PuO is actually PuO_*x*_C_*y*_.

The story of PuO was supposed to end here. However in 1990, Haschke^9^ reopen this question by claiming that the residue from the decomposition of plutonium monoxide monohydride (PuOH)^15,16^ may be the metastable PuO. Because of the extreme pyrophoric nature of the residue, even XRD measurements were not attempted. Afterwards Russian scientist Reshetnikov^10^ reported the preparation of pure PuO in gramme amounts by reacting plutonium oxychloride with calcium. But there is no any characterization of this compound.

Since this question has existed for such a long time, it is necessary to find the solution from a new point of view. In recent years, first-principles calculations have emerged as a powerful and indispensable tool for quantitatively predicting the properties of materials such as actinide oxides^17^. In addition, plutonium and its compounds are sensitive to variables such as impurity content, temperature, and pressure^1^. In particular, pressure is often used as an efficient tool to stabilize the materials or explore the new phase. Larson and Haschke^13^ already argued that PuO may be synthesized under high pressure, by noting that several lanthanide elements (La-Nd^18^, Sm^18,19^, Yb^20^) react with their higher oxides to form NaCl-type monoxides under pressure. For actinide elements, ThO was proved to be stable under pressure through first-principles calculation^21^. Therefore, in this work we employ the density-functional theory (DFT) and DFT + *U* to study the reaction (1) under pressure as well as the physical properties of PuO.

From available literature, *α*-Pu is stable up to around 40 GPa^22^, whereas no report is available on the high-pressure study of *α*-Pu_2_O_3_. In addition, for our estimate of formation enthalpy of Pu, we used the *δ*-phase, which is only 0.047 eV/atom^12^ higher than the (most stable) *α*-phase. The first-principles calculation of *α*-Pu is not a trivial task since there is a significant site-selective electronic correlation in this complicated metal^23^. Similar treatment was used in the previous literature such as the calculation of formation energies of plutonium oxides^24^.

## Methods

The electronic structure problems related to first-principles calculation were solved using the code Vienna Ab-initio Simulation Package (VASP)^25^. Within the framework of density-functional theory (DFT)^26,27^, the exchange correlation potential in the Kohn-Sham equation^27^ was described by the local-density approximation (LDA)^28^ and the generalized gradient approximation (GGA) of Perdew, Becke and Ernzerhof (PBE)^29^. Moreover, the rotationally invariant DFT + *U* approach^30^ was also utilized to capture the possible localization effect of 5*f* electrons coming from the strong electron-electron interaction. Within DFT + *U*, the “full localized limit” double counting expression and the typical parameters: *U* = 4.0 eV and *J* = 0.7 eV, are chosen here. These choices were already proved to give a reasonable description of Pu^24^ and Pu_2_O_3_^31,32^. In addition, scalar relativistic calculations were performed using spin-orbit coupling (SOC) but SOC was not included in the enthalpy calculation because it is already found that the inclusion of SOC has insignificant effect on the bulk properties^24,31–36^. For example, within GGA, inclusion of SOC increases the equilibrium lattice parameter of PuO by only 0.14%. However, GGA + SOC is used for electronic structure calculation to illustrate the spin-orbit splitting in the 5*f* states.

Furthermore, the projector augmented-wave (PAW) method^37,38^ was chosen to solve the Kohn-Sham equation. The PAW pseudopotentials for Pu and O were generated using the 6*s* ^2^7*s* ^2^6*p* ^6^6*d* ^2^5*f* ^4^ and 2*s* ^2^2*p* ^4^ valence electronic configurations, respectively. These pseudopotentials are implemented in the VASP code^25^. All computational parameters were carefully selected to yield a total energy convergence better than 1 meV/atom. Here the cut-off energy for plane wave basis set was set as 700 eV and the Brillouin zone of *δ*-Pu, PuO, and *α*-Pu_2_O_3_ are sampled by 8 × 8 × 8, 8 × 8 × 8, and 4 × 4 × 4 Monkhorst-Pack *k* point sampling mesh^39^, respectively. Here we also considered three possibilities for the magnetic states: nonmagnetic (NM), antiferromagnetic (AFM), and ferromagnetic (FM). Within the above computational schemes, the lowest-energy magnetic states of *δ*-Pu and PuO are AFM, while that of *α*-Pu_2_O_3_ is FM for pure DFT and AFM for DFT + *U*. These findings are in accord with the previous literature^24,31–36,40^.

## Results and Discussion

### Thermodynamical stability under ambient pressure

The reaction enthalpy of (1) was defined by

2 $${\rm{\Delta }}H=3{H}_{{\rm{PuO}}}-({H}_{{\rm{Pu}}}+{H}_{{{\rm{Pu}}}_{{\rm{2}}}{{\rm{O}}}_{3}}),$$

where *H*_PuO_, *H*_Pu_, and ${H}_{{{\rm{Pu}}}_{2}{{\rm{O}}}_{3}}$ are the formation enthalpy of NaCl-type PuO, *δ*-Pu, and *α*-Pu_2_O_3_, respectively. At ambient pressure, the lattice parameter of PuO, *a*_PuO_, the formation enthalpies of PuO and Pu_2_O_3_, and the reaction enthalpy ${\rm{\Delta }}H$ calculated using LDA/GGA and LDA/GGA + *U* are listed in Table 1. For comparison, the available experimental data and the previous theoretical results using self-interaction correction (SIC)^41^ and full-potential linearized augmented plane wave (FLAPW)^34^ are also shown.

**Table 1:** The lattice parameter *a*_PuO_ and formation enthalpy *H*_PuO_ of PuO, formation enthalpy of Pu_2_O_3_, ${H}_{{{\rm{Pu}}}_{2}{{\rm{O}}}_{3}}$, and the reaction enthalpy ${\rm{\Delta }}H$ defined by equation (2) under ambient pressure. For comparison, we also show the FLAPW results in the square brackets, the SIC result and the experimental values.

Method	*a*_PuO_ (Å)	${H}_{{\bf{PuO}}}^{{\boldsymbol{f}}}$ (eV/f.u.)	${H}_{{{\bf{Pu}}}_{{\bf{2}}}{{\bf{O}}}_{{\bf{3}}}}^{{\boldsymbol{f}}}$ (eV/f.u.)	$\Delta H$(eV)
LDA	4.79 [4.80^34^]	−5.37	−15.61	−0.15
GGA	4.92 [4.96^34^]	−4.82	−14.54	0.50
LDA + *U*	4.89 [4.99^34^]	−5.76	−17.13	0.41
GGA + *U*	5.03 [5.12^34^]	−5.01	−15.77	1.49
LDA + SIC	5.13^41^			
Exp.	4.96 ± 0.01^5^	−5.85 ± 0.22^12^	−18.65 ± 0.17^12^	1.10 ± 0.83
			−16.39^1^	

For *a*_PuO_, the calculated values are consistent with the previous theoretical results^34^, and GGA and LDA + *U* reproduce well the experimental data. The deviation between our work and the FLAPW results^34^ may due to the different magnetic states and different schemes of DFT + *U*. The lattice parameters predicted by DFT + *U* are larger than those from pure DFT. This is because DFT + *U* scheme favours the localization of Pu 5*f* electrons, consequently Pu 5*f* electrons participate less to the bonding which leads to an increase of lattice parameter. In addition, since the overbinding is usually found in the LDA, the LDA underestimate the lattice parameter. Overall, GGA and LDA + *U* gives a better description while pure LDA underestimates the lattice parameter and GGA + *U* overestimates it. Similar deviations of LDA/GGA and LDA/GGA + *U* have been found in their description of Pu^24,40^ and PuO_2_^24,42,43^.

**Figure 1:**
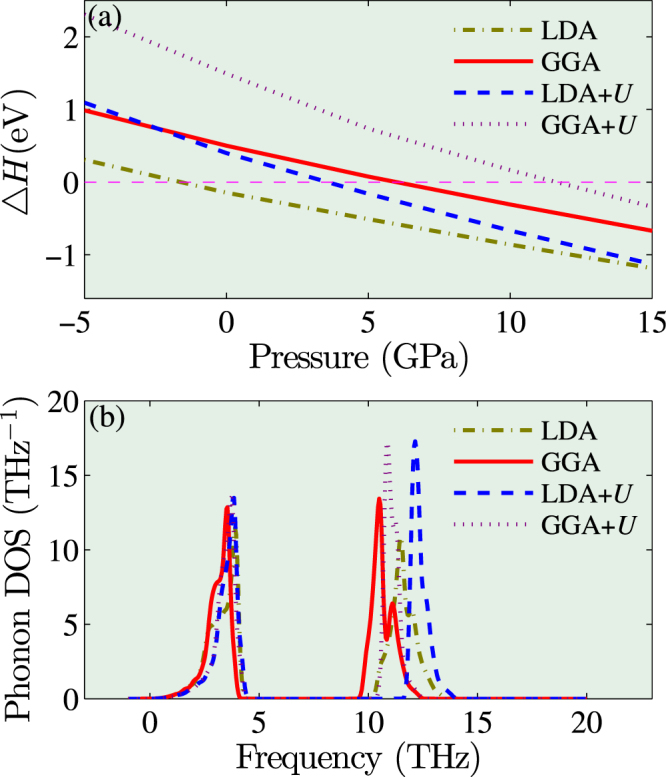
Thermodynamical and dynamical stability of PuO. (**a**) The reaction enthalpy ${\rm{\Delta }}H$ and (**b**) the phonon density of states of PuO at 15 GPa obtained from LDA/GGA and LDA/GGA + *U*.

The calculated formation enthalpies of PuO and Pu_2_O_3_ are in reasonable agreement with the experimental values. This indicates that our calculations are consistent and reliable. The larger formation enthalpies found in LDA(+*U*) with respect to GGA are consistent with the overbinding in LDA(+*U*). For same reason, the reaction (1) is more exothermic in LDA(+*U*) than in GGA(+*U*). Under ambient pressure, the calculated reaction enthalpy is positive only except the LDA result. However, the lattice parameter of PuO predicted by LDA deviates the experiment by 0.17 Å, which corresponds to a larger negative pressure ~ −13 GPa. Similar underestimate by LDA is also found in the other plutonium compounds^24,40,42,43^. We will see that ${\rm{\Delta }}H$ is positive at a small negative pressure of ~ −1 GPa (see Fig. 1(a)). Thus it is reasonable to reckon the modified value of $\Delta H$ in LDA by enlarging the cell as positive. Overall, our first-principles calculation predicted that PuO is thermodynamically unstable under ambient pressure. As discussed in the introduction, this finding agrees with the high temperature XRD^6^ and XPS-AES^13^ experiment, whereas disagrees with the recent preparation experiment^10^.

### Thermodynamical and dynamical stability under high pressure

In Fig. 1(a) we plot the reaction enthalpy as a function of pressure which ranges from −5 GPa to 15 GPa. It is clear that the reaction enthalpy becomes negative when pressure is increased above a critical value *P*_c_. That is to say, PuO is thermodynamically stable above this critical pressure *P*_c_. From Fig. 1(a), the critical pressures predicted by GGA and GGA + *U* are 6 and 12 GPa, respectively. Thus it is likely that *P*_c_ lies within this range 6–12 GPa.

In the following, we will take a value of 15 GPa as the pressure after the transition. The dynamical stability of PuO under pressure is examined by the phonon calculations. Figure 1(b) plot the phonon density of states (DOS) of PuO using LDA/GGA and LDA/GGA + *U*. No imaginary frequency was found for the entire Brillouin zone. The large frequency gap observed in Fig. 1(b) was attributed to the large mass ratio between Pu and O, i.e., Pu atoms contribute the lowest acoustic modes (0–5 THz) and O atoms contribute the higher modes (10–13 THz). In a word, PuO is dynamically stable in rocksalt structure.

### Electronic structure

**Figure 2:**
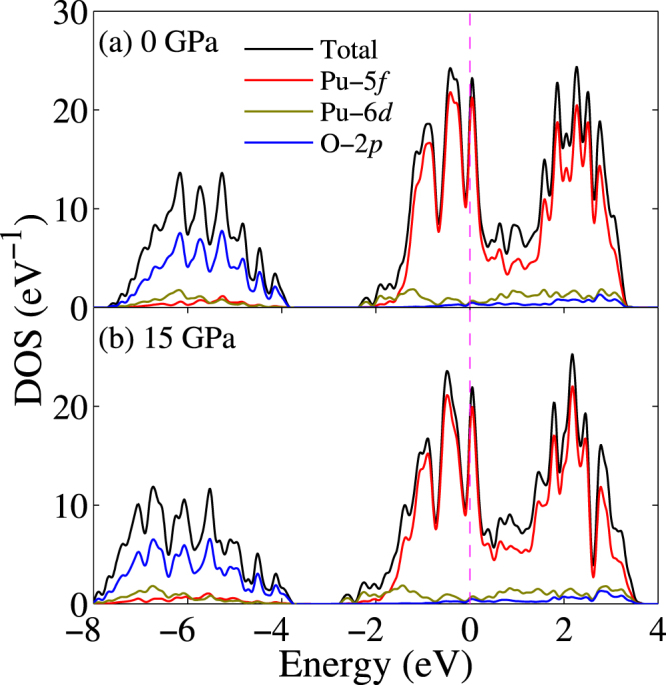
Total and projected density of states within the GGA + SOC formalism under pressure of (**a**) 0 GPa and (**b**) 15 GPa. The Fermi level is denoted by the dashed line.

To obtain insights into the stabilization of PuO under pressure, we investigated and compared the electronic structures at 0 GPa and 15 GPa. The total density of states (DOS) and projected DOS (pDOS) for Pu-5*f*, Pu-6*d* and O-2*p* states are plotted in Fig. 2. Note that SOC is included in the following. Apparently, PuO is metallic, which is consistent with the previous theoretical result^34^.

It was well-known that Pu-6*d* orbital is quasi-degenerate with Pu-5*f* orbital and thus the hybridization between Pu-5*f* and Pu-6*d* states are very strong in the plutonium compounds. This feature is apparent in Fig. 2 and those bands could be denoted by Pu-5*f*/6*d* states. We can also see that the conduction bands and the semicore bands (energy bands between −8 eV and −4 eV) are mainly contributed by Pu-5*f*/6*d* and O-2*p* states, respectively. The sharp-peak feature of Pu-5*f* pDOS indicates the localization of the Pu-5*f* states. In the conduction and semicore bands, the shape of Pu-5*f* and Pu-6*d* pDOS follow that of O-2*p*, which implies that there is a considerable hybridization between O-2*p* and Pu-5*f*/6*d* states. This also indicates the presence of Pu-O bond. Through Bader charge analysis^44^, the charge transfer from Pu to O is evaluated to be about 1.3 *e*, which approximates half of that in PuO_2_^43^. This suggests the formal oxidation state of Pu in PuO is indeed II.

**Figure 3:**
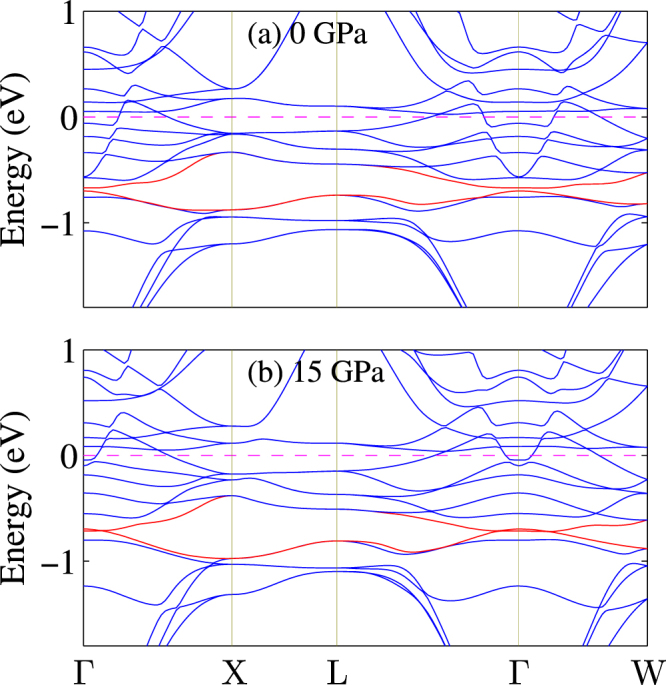
Band structures around the Fermi level within the GGA + SOC formalism under pressure of (**a**) 0 GPa and (**b**) 15 GPa. The Fermi level is denoted by the dashed line. The red lines show the crossing band under pressure.

Next let us focus the effect of pressure. Under pressure, the bandwidth of conduction bands and semicore bands is increased and the band gap among them is decreased. This implies that the Pu-5*f* states become relatively itinerant and participate more and more to the hybridization and bonding. To clarify this, let us make a close look at the band structures. The band structures of PuO along ${\rm{\Gamma }}$-X-L-${\rm{\Gamma }}$-W directions are shown in Fig. 3. The band shift under pressure is highlighted and it is clear that pressure forces Pu-5*f*/6*d* bands and O-2*p* to mix together at ${\rm{\Gamma }}$ point. So we conclude that hybridization among Pu-5*f*, Pu-5*d*, and O-2*p* serves as the dominant stabilizing factor.

### Optical properties

For the synthesis of LnO (Ln = Lanthanide) under high pressure^18^, the mixture of Ln and Ln_2_O_3_ were introduced into a compressible gasket apparatus of the belt type calibrated by using the polymorphic transition of bismuth and barium. After being unloaded, the products were identified from XRD and chemical analysis. For the synthesis of PuO, modern *in situ* diamond-anvil cell x-ray/laser-heating experiment may provide more information than the older methods. For example, XPS could also be used to identify the phase of PuO. In particular, optical detection is often used in the characterization of new metallic phase in the high pressure experiment. For example, the important evidence of the metallic hydrogen at 495 GPa is the high reflectivity^45^.

**Figure 4:**
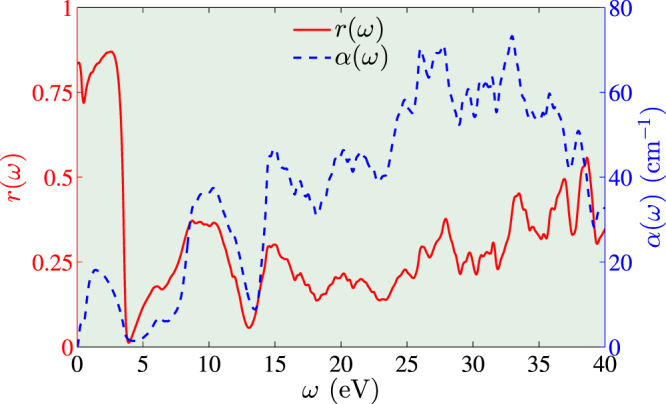
The calculated reflectivity $r(\omega )$ (left) and absorption coefficient $\alpha (\omega )$ (right) of PuO at 15 GPa using the GGA + SOC scheme.

Here we also calculate the optical properties of PuO including the reflectivity and absorption coefficient, as shown in Fig. 4. The optical conductivity is computed from the Kubo-Greenwood formula^46,47^ within the independent-particle approximation. And then the optical properties are calculated using Kramers-Kronig relation. The technical details are described in ref.^48^. The peak observed at 2 eV is mainly contributed by inter-band transition, i.e., the transition from Pu-5*f* states at the bottom of the conduction band to the Pu-5*f* states at the top of the conduction band. And the peaks at 6 and 10 eV are contributed from the O-2*p* states to the conduction band. The peaks at higher energy are due to transition from semicore states to the conduction band. This information is useful for the optical detection of the plutonium monoxide in the high pressure experiment.

## Conclusion

In this work, we have calculated the reaction enthalpy of reaction Pu_2_O_3_ + Pu → 3 PuO in the framework of DFT and DFT + *U*. The results suggest that PuO is thermodynamically unstable at ambient pressure and probably decompose into *α*-Pu_2_O_3_ and Pu. However, PuO may be stabilized by the other environment variables such as impurity and temperature. Here we find that PuO is likely to exist at the pressure above 12 GPa by calculating the reaction enthalpies and phonon density of states under high pressure. The origin of the stabilization under pressure lies in the delocalization of Pu-5*f* states and the increasing hybridization between Pu-5*f*/6*d* and O-2*p* states. Furthermore, the optical properties of PuO are also predicted and may provide a useful reference for future experiment.
